# Gut Microbiota and Clinical Manifestations in Thai Pediatric Patients with Attention-Deficit Hyperactivity Disorder

**DOI:** 10.3390/jpm14070739

**Published:** 2024-07-10

**Authors:** Jittraporn Panpetch, Komsan Kiatrungrit, Siriporn Tuntipopipat, Sithichoke Tangphatsornruang, Wuttichai Mhuantong, Nalinee Chongviriyaphan

**Affiliations:** 1 Doctoral Program in Nutrition, Faculty of Medicine Ramathibodi Hospital and Institute of Nutrition, Mahidol University, Bangkok 10400, Thailand; jittraporn.pan@student.mahidol.ac.th; 2 Department of Psychiatry, Faculty of Medicine Ramathibodi Hospital, Mahidol University, Bangkok 10400, Thailand; komsan.kia@mahidol.ac.th; 3 Institute of Nutrition, Mahidol University, Nakhon Pathom 73170, Thailand; siriporn.tun@mahidol.ac.th; 4 National Center for Genetic Engineering and Biotechnology (BIOTEC), Thailand Science Park, Paholyothin Rd., Klong Nueng, Klong Luang, Pathum Thani 12120, Thailand; sithichoke.tan@biotec.or.th (S.T.); wuttichai.mhu@biotec.or.th (W.M.); 5 Division of Nutrition, Department of Pediatrics, Faculty of Medicine Ramathibodi Hospital, Mahidol University, Bangkok 10400, Thailand

**Keywords:** attention-deficit hyperactivity disorder, ADHD, gut microbiota, gut-brain axis

## Abstract

Attention-deficit hyperactivity disorder (ADHD) is a neurodevelopmental disorder potentially linked to gut dysbiosis. This comparative cross-sectional study profiled the gut microbiota in 24 treatment-naïve Thai children diagnosed with ADHD and 24 healthy ones matched by age and gender (median age: 7 years). Fecal microbial compositions were genetically analyzed using 16s rRNA gene amplicon sequencing. The study findings indicated no statistically significant differences in microbial diversity between groups, although Firmicutes and Actinobacteria appeared dominant in both groups. Moreover, ADHD patients exhibited enrichment in *Alloprevotella*, *CAG-352*, *Succinivibrio*, and *Acidaminococcus* genera, while healthy controls had higher levels of *Megamonas*, *Enterobacter*, *Eubacterium hallii*, and *Negativibacillus* genera. Spearman correlation analysis demonstrated a significant positive association between *CAG-352* and inattention and hyperactivity/impulsivity scores, whereas the *Eubacterium hallii* group and *Megamonas* exhibited negative correlations with these symptomatology domains. Beta-carotene intake was associated with the *Eubacterium hallii* group and *Succinivibrio*: likewise, vitamin B2 intake was associated with *Alloprevotella*. Additional research should aim to elucidate the underlying mechanisms influencing clinical biomarkers that signify alterations in specific gut microbiome profiles linked to ADHD.

## 1. Introduction

Attention deficit hyperactivity disorder (ADHD) is currently the most common childhood-onset neurodevelopmental disorder [1]. ADHD is characterized by 3 clusters of developmentally inappropriate and impairing clinical manifestations: inattention, hyperactivity, and impulsivity. Based on the types of symptoms, the Diagnostic and Statistical Manual of Mental Disorders, Fifth Edition (DSM-5) mainly categorizes ADHD into three presentations: predominantly attentive presentation, predominantly hyperactive/impulsive presentation and combined presentation [1,2,3]. A recent meta-analysis study revealed the estimated data of worldwide ADHD prevalence among school-aged children, with a rate of approximately 7.6% [2]. In Thailand, a national survey of ADHD estimated its prevalence among Thai primary school children at 8.1% in 2012 [4]. The global prevalence of ADHD is increasing in society and involves a very high cost to the individual and society. ADHD might be a life-long issue for many patients treated inadequately. At least half of children with ADHD have symptoms persisting into adolescence and adulthood. These symptoms will manifest conduct disorders, failed relationships, workplace underachievement, substance abuse, and low self-esteem. Moreover, there are many problematic impacts on families and patients, including reduced social, academic, and occupational functioning, and overall quality of life [1,5].

Similar to all complex disorders, a single risk factor is insufficient to explain the etiology of ADHD. It has been shown that ADHD results from multifactorial factors such as genetic, environmental, and neurobiological interactions [6]. Multiple causative factors including gestational age, delivery mode, type of feeding, diet, antibiotic usage, epigenetics, infection as well as neurotransmission system dysfunctions may influence the risk of ADHD development and the variation of ADHD manifestations [7,8]. Nonetheless, although the precise mechanisms underpinning ADHD pathogenesis remain elusive, emerging evidence points towards a potential association between ADHD symptoms and alterations in gut microbiota [9]. Numerous investigations underscore the pivotal role of gut microbiota in the bidirectional gut-brain axis communication, influencing metabolic processes, inflammatory responses, the hypothalamic-pituitary-adrenal axis, and neurotransmitter systems [9,10,11]. The gut microbiota exerts neurobiological influence by modulating neurotransmitter levels essential for cognitive and emotional regulation, including dopamine and serotonin [12]. Despite serotonin’s primarily cerebral synthesis, a considerable portion originates in the gut [13]. Established associations between the gut microbiome, neurotransmitters, and neuropsychiatric disorders have been demonstrated by animal studies showing microbiome alterations related to anxiety and social behavior, with concomitant neurotransmission changes in relevant brain regions [14,15,16,17,18,19,20,21,22]. In humans, links between microbiome composition and emotion regulation have been discerned in relation to its capacity to release dopamine and serotonin [23,24]. While the relationship between the gut microbiome and executive function (EF) remains less elucidated in humans, evidence from rodent studies supports a dopamine-mediated influence on EF, further linking the gut microbiome to EF-related behaviors [25,26]. Additionally, the association between the gut microbiome and neuropsychiatric disorders like Autism Spectrum Disorder (ASD), characterized by impaired EF, accentuates its potential impact on EF and emotion regulation [27,28,29,30,31,32,33,34]. Due to the shared symptoms between ADHD and other neuropsychiatric conditions, notably ASD, investigating the involvement of the gut microbiome in ADHD could shed light on its underlying mechanisms and potential treatments [35,36]. 

Numerous studies have employed 16S ribosomal RNA (rRNA) sequencing to investigate the relationship between bacterial composition and the pathological mechanisms of ADHD [37,38,39,40,41]. Aarts et al. identified elevated levels of Actinobacteria and *Bifidobacterium* in individuals with ADHD [37], whereas Jiang et al. reported decreased levels of *Faecalibacterium*, *Dialister*, and *Sutterella*, without significant differences in overall microbial diversity [38]. Prehn-Kristensen et al. found significant differences in both alpha and beta diversity, noting increased levels of *Bacteroidaceae* and *Neisseriaceae* in ADHD patients [40]. In addition, gene-set enrichment analysis linked ADHD to *Desulfovibrio* and *Clostridiales* and revealed variations in *Bacteroides* and *Sutterella* between ADHD patients and healthy controls [41]. *Bifidobacterium*, involved in the dopamine reward system, along with genera such as *Faecalibacterium*, *Anaerotaenia*, and *Gracilibacter*, has been found to be associated with attention deficits, suggesting that gut microbiota dysbiosis may contribute to ADHD pathophysiology [39]. 

However, findings on alpha diversity remain inconclusive due to study heterogeneity. These inconsistencies, influenced by demographic factors, medication use, and nutritional variations, underscore the need for further research to elucidate the underlying mechanisms. Therefore, this study aimed to determine gut microbiome profiles in treatment-naïve Thai ADHD children. We proposed that treatment-naïve Thai children with ADHD may exhibit distinct gut microbial compositions based on their ADHD symptomatology compared to healthy counterparts. In addition, this study was conducted to delineate differential patterns of gut microbiome and dietary intakes between ADHD children and the controls.

## 2. Subjects and Methods

### 2.1. Study Design and Participants

This study was carried out as a comparative cross-sectional study approved by the Institutional Review Board (IRB) of the Faculty of Medicine Ramathibodi Hospital, Mahidol University, and the IRB of Buddhasothorn Hospital in Thailand. The procedures were conducted according to the 1996 Declaration of Helsinki. All participants who met the criteria for recruitment and their parents were fully informed of the purpose, procedures, and hazards involved in the study. Written informed consent was obtained from the participants and their parents before enrollment. Participants in the ADHD group were recruited from eligible patients aged between 6 and 12 years who were treated in the outpatient department of the Division of Child and Adolescent Psychiatry at Buddhasothorn Hospital in Thailand. The inclusion criteria for ADHD patients were as follows: (1) a clinical diagnosis of ADHD according to the DSM-5 classification system assessed by a certified child and adolescent psychiatrist; (2) having scored above the standard clinical cutoffs for ADHD symptoms on the Thai version of Swanson, Nolan, and Pelham Rating Scale-IV (SNAP-IV) [42]; and (3) having not received any medication yet. We excluded patients who had: (1) been diagnosed with primary psychiatric disorders or significant physical illness such as intellectual disability, genetic disorders, cerebral palsy, autism, neuromuscular diseases, epilepsy, brain trauma, encephalitis, or chronic atopic diseases; (2) taken any medications or probiotics within the two months before the fecal collection; and (3) obesity. The control group comprised healthy children aged between 6 and 12 years studying in regular classes at primary schools. They did not have childhood developmental disorders as confirmed by the Thai version of SNAP-IV criteria. In addition, children in the control group were not children diagnosed with other comorbid conditions. They had no known behavioral, academic, or social difficulties. As with the ADHD group, those currently taking medications or probiotic supplements were excluded. 

Demographic data, including age, gender, home type, early feeding practices, mode of delivery, gestational age (and premature birth status if applicable), birth weight, and health behaviors such as bowel habits, were reported by the children’s parents or guardians using study-specific questionnaires. Bowel habits were assessed using the Bristol Stool Chart, which was employed to identify stool shapes and types and functioned as a diagnostic tool for constipation [43]. For dietary information, a nutritionist interviewed the parents or guardians to record dietary consumption during the month before the visit, utilizing semi-quantitative food frequency questionnaires (FFQs). Additionally, three non-consecutive 24-h dietary recalls (two weekdays and one weekend day) documented the menu, ingredients, and quantities of each meal, including breakfast, mid-morning snack, lunch, mid-afternoon snack, dinner, and after-dinner snack, before fecal sample collection. For participants under ten years of age, dietary intake information was provided by their parents. Energy, macronutrient, and micronutrient intakes were calculated using the INMUCAL-Nutrients software V.4.0 from the Institute of Nutrition, Mahidol University [44]. Due to the absence of prebiotic and probiotic information in the INMUCAL-Nutrients software, the intakes of these nutrients were estimated using nutrition labels on food products.

### 2.2. Sample Collection and 16S rRNA Gene Amplicon Sequencing

The stool samples were collected from the children assisted by their parents, kept in a sterile plastic tube with a tightly closing lid, stored in an insulated bag, and then immediately frozen at home. The samples were held in an icebox, transported to the hospital laboratory unit, and stored at −80 °C for further analysis. According to the manufacturer’s instructions, total fecal bacterial DNA was extracted from 200 mg of feces using the QIAamp^®^Fast DNA stool mini kit (Qiagen, Hilden, Germany). DNA concentration was measured using the Qubit^®^2.0 Fluorometer (Life Technologies, Carlsbad, CA, USA) and stored at −20 °C before analysis. 

The bacterial 16S rRNA gene V3-V4 region was amplified with a barcoded primer set using 338FACTCCTACGGGAGGCAGCAG and 806R GGACTACHVGGGTWTCTAAT. Approximately 4 μL of 5 × FastPfu Buffer, 0.8 μL of 2.5 mM deoxyribonucleotide triphosphates (dNTPs), 0.8 μL of 5 μM reverse primer, 0.4 μL of FastPfu Polymerase, and a 10-ng template were employed in the polymerase chain reaction (PCR). The PCR cycling conditions comprised an initial denaturation step of 3 min at 95 °C, followed by 27 cycles of denaturation at 95 °C for 30 s, annealing at 55 °C for 30 seconds, extension at 72 °C for 45 s, and a final extension step of 10 min at 72 °C. Subsequently, after the initial PCR clean-up procedure, the samples underwent amplification with dual indices employing an Illumina sequencing adapter integrated with the Nextera XT Index Kit (Illumina Inc., San Diego, CA, USA). The resulting Amplicon Library was pooled, normalized, supplemented with PhiX Control, and subjected to sequencing. Sequencing of the 16S rRNA was executed utilizing the Illumina MiSeq platform, employing the TruSeqTM DNA Sample Prep Kit (Illumina Inc., San Diego, CA, USA) [38]. 

### 2.3. Bioinformatics and Statistical Analysis

The sequence data analysis commenced with the sequence quality control (QC) of raw paired-end (PE) sequences using FASTP [45]. Cleaned PE sequences were merged into single-end reads utilizing FLASH [46]. The ASV table was then constructed via DADA2 [47] and implemented in QIIME2 version 2023.2 [48]. The alpha diversity index and the rarefaction curve of the saturated microbiome were calculated based on the subsampling method of 43,798 reads per sample. Bacterial profiles were classified using consensus blast against the Silva database, version 132 (latest release on 2 November 2020), applying a percent identity cutoff of 80%. The gut microbiome was investigated either within-sample diversity (alpha diversity) or between-sample diversity (beta diversity). To determine alpha diversity, three metrics, including richness, Chao1 index, and Shannon index, were computed and compared using the Kruskal-Wallis test. Beta diversity was assessed using Principal Coordinates Analysis (PCoA) plots based on Weighted UniFrac, Unweighted UniFrac, and Bray-Curtis distances. Group differences were evaluated for significance with pairwise permutational multivariate analysis of variance (PERMANOVA) using 999 random permutations. Additionally, microbiome community comparisons and cladograms were conducted using linear discriminant analysis effect size (LEfSe). Functional abundances and KEGG pathway predictions were performed using the Phylogenetic Investigation of Communities by Reconstruction of Unobserved States (PICRUSt2) pipeline in the QIIME2 plugin. Taxonomic and functional profiles generated by PICRUSt2 were analyzed and visualized using Statistical Analysis of Taxonomic and Functional Profiles (STAMP). Basic statistical analyses were conducted using SPSS version 19.0 software (SPSS Inc., Armonk, NY, USA). Depending on the data type, categorical and continuous variables between groups were compared using chi-squared, nonparametric, and Student’s *t*-tests. Correlations between bacterial DNA concentration and the average scores on the inattention and hyperactivity/impulsivity subsets, as well as nutrient consumption, were analyzed using Spearman’s rank test.

## 3. Results

The study was performed between March 2019 and March 2022 in Chachoengsao, Thailand. Among 48 participants enrolled in the study, 24 participants were treatment-naïve patients with ADHD referred to the outpatient unit at the Division of Child and Adolescent, Department of Psychiatry, Buddhasothorn Hospital, Chachoengsao, and 24 were age- and sex-matched healthy children recruited from children with normal development, studying in regular classes at primary schools. 

The median age of the participants was 7 years old. Most participants were male. There were no significant differences in gender, age, anthropometric data, type of delivery, gestational age, birth weight, birth length, head circumference, continued breastfeeding time, and constipation. Accordingly, there was a significantly higher severity score on the SNAP-IV scale rated by parents and teachers in the ADHD group than in the healthy group. In the ADHD group, 75% of participants had a predominantly combined type of ADHD, 20.8% predominantly inattentive ADHD, and 4.2% had predominantly hyperactive-impulsive ADHD. The general characteristics of the study participants are presented in Table 1.

**Figure 1:**
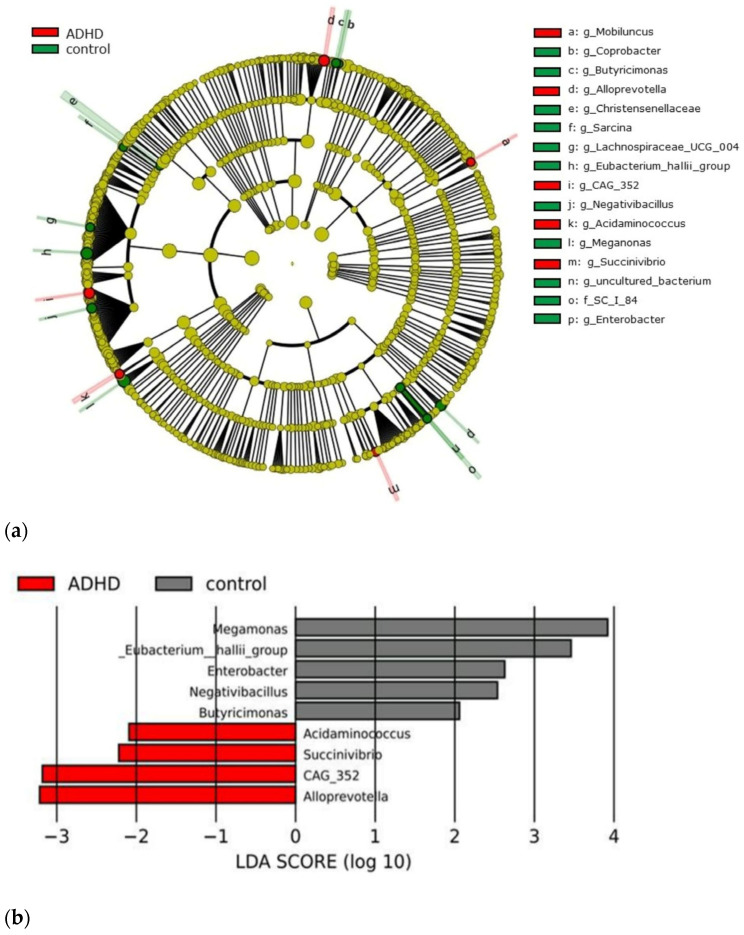
A cladogram illustrating differentially abundant taxa in the gut microbiota is presented. (**a**) Genus-level taxon features were identified by LEfSe with an LDA score greater than 2, comparing treatment-naïve Thai children with attention-deficit hyperactivity disorder (ADHD) to age- and sex-matched healthy controls. (**b**) Red bars indicate taxa with significantly higher expression in children with ADHD, while grey bars represent those in healthy controls. Group differences were assessed using the Kruskal-Wallis test, with a significance threshold of *p* < 0.05. LDA denotes linear discriminant analysis, and LEfSe represents linear discriminant analysis effect size.

Based on the sequencing data, there were 5,284,081 sequencing reads clustered into 2,078 operational taxonomic units (OTUs), 28 phyla, 53 classes, 134 orders, 231 families, 499 genera, and 620 species. The relative abundance at the phylum level in ADHD patients exhibited a dominance of Firmicutes (61.96%), Actinobacteria (25.71%), Bacteroidetes (9.75%), and Proteobacteria (1.62%). The relative abundances of these major phyla in the control group were largely similar to the ADHD group. Therefore, the gut microbiome profiles of the ADHD patient group and the control group were similar. A LEfSe plot was developed and presented for relative abundance comparison to distinguish the genus variation between groups. The relative abundance of *Alloprevotella*, *CAG-352*, *Succinivibrio*, and *Acidaminococcus* was predominant in the ADHD group. On the other hand, the relative abundance of *Megamonas*, *Eubacterium hallii* group, *Enterobacter*, *Negativibacillus*, and *Butyricimonas* become the notable genus in the healthy control group. The LEfSe plot is shown in Figure 1.

**Figure 2:**
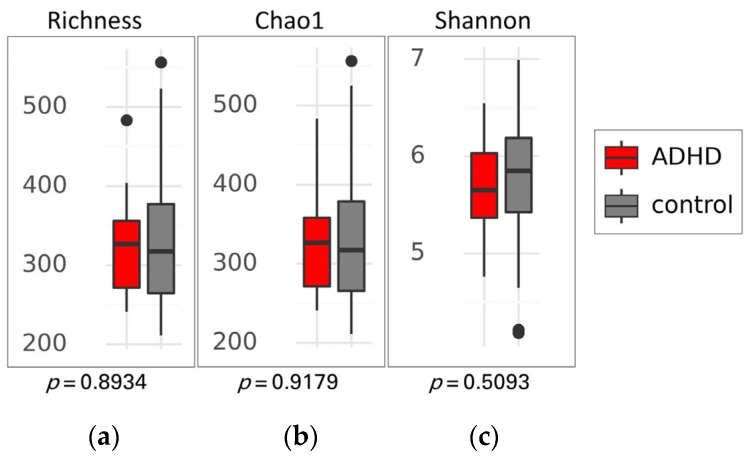
Alpha diversity of gut microbiota between patients with attention-deficit hyperactivity disorder (ADHD) and healthy children (control). Box plots show the richness (**a**), Chao1 index (**b**), and Shannon index (**c**), along with their respective *p*-values. Differences between groups were analyzed using the Kruskal-Wallis test. The boxes display the interquartile range (IQR) from the 25th to the 75th percentiles, with a line representing the median. Notches indicate the 95% confidence interval for the median. Whiskers extend to 1.5 times the IQR, and any potential outliers are depicted as dots.

**Figure 3:**
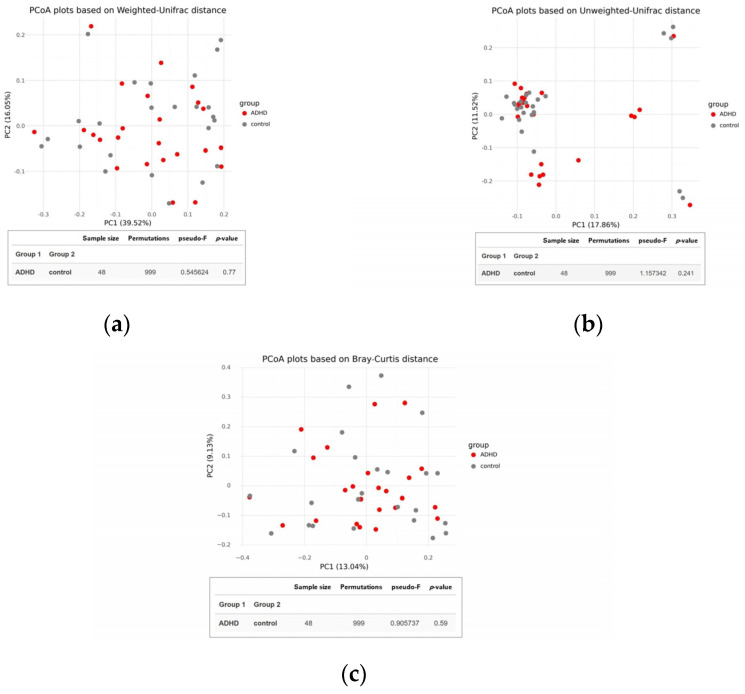
Beta diversity of the gut microbiota between children with attention-deficit hyperactivity disorder (ADHD) and healthy controls. PCoA plots were constructed using (**a**) Weighted UniFrac, (**b**) Unweighted UniFrac, and (**c**) Bray-Curtis distances. Group differences were evaluated through permutational multivariate analysis of variance (PERMANOVA).

There was no significant difference in alpha diversity between the two groups (Figure 2), suggesting that the composition of gut microbiota was similar in both ADHD and healthy children. This finding was supported by the PCoA plot and statistical analysis using permutational multivariate analysis of variance (PERMANOVA) based on Weighted UniFrac, Unweighted UniFrac, and Bray-Curtis distances, which showed no distinct clustering with *p*-values of 0.770, 0.241, and 0.590, respectively (Figure 3). 

The scores in the two domains of ADHD symptoms, inattention and hyperactivity/impulsivity, and the relative abundance of 9 bacterial taxons identified as showing a significant difference by the LEfSe plot were used for investigating the association between gut microbiota and clinical manifestation of ADHD. The analyses revealed that the relative abundance of *Eubacterium hallii* group, *Megamonas*, and *Negativibacillus* was negatively correlated with inattention scores reported by parents and teachers, whereas *CAG-352* and *Alloprevotella* levels were positively correlated with these scores. Similarly, in the domain of hyperactivity/impulsivity symptoms, the result showed that the relative abundance of the *Eubacterium hallii* group, *Megamonas*, and *Butyricimonas*, was negatively correlated with hyperactivity/impulsivity scores reported by parents and teachers. On the other hand, the *CAG-352* level was the only genera that positively correlated with these modules (Table 2).

Based on semi-FFQ data designed to assess habitual diet based on particular food items, there was no significant difference in types and portions of dietary intake between ADHD patients and healthy children. The 3-day food record data to determine nutrient intake details showed no significant differences in the daily energy and macronutrient intake. The percentage of the energy distribution of macronutrients in both groups was 49:19:32 for carbohydrate, protein, and fat, respectively, which was within the recommendations for Thai school-age children from Thai Dietary Reference Intakes (DRIs) [49,50]. However, there were significant differences in micronutrient intakes. The median intake of copper, vitamin B2, and vitamin B3 was significantly lower in the ADHD group compared to the control group. 

Finding this significance led to the analysis of daily micronutrient intake categorized by adequate consumption among participants. According to the recommended energy and nutrient intake values in Southeast Asian countries with the Estimated Average Requirement (EAR) or the Average Intake (AI) for children aged 6–12 years, ADHD patients and healthy children were reclassified into adequacy or inadequacy groups [51]. There was no statistically significant difference in the proportion of ADHD patients and healthy children between the adequacy and inadequacy groups for each micronutrient (Table 3). Despite no statistical significance of DRI compliance analysis, there was a difference in participant proportions by the adequacy of vitamin B3 consumption between ADHD patients and healthy counterparts. Vitamin B3 intake analysis shows that 95.8% of healthy children and 79.2% of ADHD patients had adequate vitamin B3 consumption (*p* = 0.081).

Furthermore, a statistically significant correlation between nutrient consumption and gut microbiome profiles was detected, as shown in Table 4. Specifically, there was a positive correlation noted between beta-carotene intake and the relative abundance of the *Eubacterium hallii* group, alongside a negative correlation with the relative abundance of *Succinivibrio*. Conversely, a negative correlation was identified between vitamin B2 intake and the relative abundance of the *Alloprevotella* genera.

**Figure 4:**
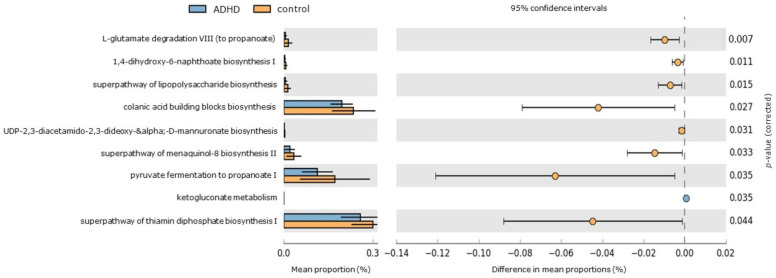
Differentially abundant KEGG pathways in ADHD and healthy control groups. An extended error bar plot was generated to visualize significantly different KEGG pathways identified through PICRUSt2 analysis. The left sidebar plots illustrate the mean proportions of each KEGG pathway. On the right, dot plots depict the differences in mean proportions between the specified groups, with only those showing *p*-values < 0.05 (Welch’s test) displayed.

PICRUSt2 analysis was utilized to predict functional KEGG pathways of gut microbiota [52], aiming to evaluate whether taxonomic variations correlate with potential functional changes (Figure 4). Among the healthy control group, significant enrichment was observed in 8 pathways. These pathways include functions related to short-chain fatty acid “propionate” production (such as pyruvate fermentation to propanoate I and L-glutamate degradation VIII to propanoate), thiamin synthesis in bacteria (superpathway of thiamin diphosphate biosynthesis), biosynthesis of polyanionic heteropolysaccharides containing repeat units with D-glucose, L-fucose, D-galactose, and D-glucuronate sugars (colanic acid building blocks biosynthesis), lipopolysaccharide biosynthesis (superpathway of lipopolysaccharide biosynthesis and UDP-2,3-diacetamido-2,3-dideoxy-α-D-mannuronate biosynthesis), and menaquinone biosynthesis (including 1,4-dihydroxy-6-naphthoate biosynthesis I and superpathway of menaquinol-8 biosynthesis II). In contrast, the pentose phosphate pathway (associated with ketogluconate metabolism) was significantly elevated in the ADHD group.

## 4. Discussion

This comparative cross-sectional study represents the inaugural situational analysis of the gut microbiome, employing a 16S rRNA sequencing platform to delineate fecal microbiota composition in treatment-naïve Thai children with ADHD and their healthy counterparts. Our investigation revealed no significant disparity in gut microbial diversity, encompassing both alpha and beta diversity, between the two groups. Notably, the composition of the gut microbiota exhibited similarity across both groups, with Firmicutes emerging as the predominant phylum, followed sequentially by Actinobacteria, Bacteroidetes, and Proteobacteria. This finding aligns with prior research on longitudinal gut microbiota dynamics during childhood conducted in the Netherlands. Houtman et al. [53] explored gut microbial diversity in 193 healthy mother-infant pairs over the initial 12 years of life. Their study highlighted Firmicutes as the most prevalent phylum among participants aged 6 to 10 years, which corresponds well with the life stage of our participants.

Moreover, our investigation identified a heightened relative abundance of the genera *Alloprevotella*, *CAG-352*, *Succinivibrio*, and *Acidaminococcus* in the ADHD group compared to healthy children. Conversely, the relative abundance of the genera *Megamonas*, *Eubacterium hallii* group, *Enterobacter*, and *Negativibacillus* was notably higher in healthy children. This analysis offers valuable insights into the potential correlation between gut microbiota composition and ADHD symptomatology, particularly concerning inattention and hyperactivity/impulsivity. Correlation analysis indicated a significant positive relationship with the *CAG-352* genera. In contrast, the *Eubacterium hallii* group and the *Megamonas* genera exhibited negative associations with inattention and hyperactivity/impulsivity scores reported by parents and teachers. Additionally, the level of the *Eubacterium hallii* group in children with ADHD was significantly lower than that in healthy children.

According to the results of this study, it is possible that the *CAG-352* genus could be a significant contributing factor associated with the symptoms of ADHD. Although no previous studies have shown a direct link between the *CAG-352* genus and the pathophysiology of ADHD, there are studies showing that the *Ruminococcaceae* family, which includes the *CAG-352* genus, was correlated with symptoms of inattention in ADHD and various other psychiatric disorders such as autism, bipolar disorder, anxiety, depression, and schizophrenia [54,55,56]. Szophinska-Tokov J, et al. reported results suggesting that the *Ruminococcaceae* family was related to inattention symptoms and might have a potential role in ADHD conditions [57]. Additionally, a study conducted by Tengeler AC, et al. demonstrated that introducing the gut microbiota of ADHD-afflicted individuals into germ-free mice significantly heightened anxious behavior [58]. Notably, the relative abundance of the *Ruminococcaceae* family was linked to abnormal emotional and behavioral symptoms associated with mental health conditions, including ADHD. Further investigation is required to explore the correlation between the abundance of the *CAG-352* genus and ADHD. 

The influence of dietary intake on both the composition and functionality of the gut microbiota warrants consideration. Our investigation showed no discrepancy in dietary intake between individuals diagnosed with ADHD and their healthy counterparts. However, individuals with ADHD exhibited markedly lower median intake levels of copper, beta-carotene, vitamin B2, and vitamin B3 compared to the healthy group. In particular, there was a positive association between beta-carotene intake and the increased prevalence of the *Eubacterium hallii* group, predominantly found in healthy children, while simultaneously exhibiting a negative correlation with *Succinivibrio*, which displayed higher abundance in individuals with ADHD. Moreover, there was a negative correlation of vitamin B12 intake with *Alloprevotella*, a genus more abundantly present in individuals diagnosed with ADHD.

Beta-carotene, a fat-soluble provitamin A carotenoid, is renowned for its antioxidant and anti-inflammatory properties [59]. The evidence suggests that cognitive deficits and disruptions in sensory processing, progressive maturation, and social behavior may be linked to low vitamin A levels in children with ADHD [60]. Additionally, studies on gut microbiota have underscored the role of beta-carotene in promoting gut health through immunoglobulin A production, contributing to gut immune system maturation, and mitigating gut dysbiosis in inflammatory conditions [59,61,62,63,64]. Likewise, riboflavin or vitamin B2 plays a pivotal role in antioxidant effects as a precursor of flavin nucleotide and flavin adenine dinucleotide, coenzymes of glutathione reductase implicated in scavenging reactive oxygen species for cellular homeostasis [65,66]. Furthermore, the involvement of vitamin B2 in the kynurenine pathway, in intrinsic neuroprotection against glutamate excitotoxicity [67], and its impact on tryptophan catabolism underscores its relevance to ADHD development [68].

Previous research has indicated that short-chain fatty acids (SCFAs), such as butyrate, propionate, and acetate, produced through anaerobic bacterial fermentation of dietary fiber in the intestine, are critical microbial metabolites linking gut microbial composition alterations to brain dysfunction [69,70,71,72,73]. These SCFAs can cross the blood-brain barrier, serve as primary energy sources for glial cells and neurons, and play a vital role in brain development, particularly in early life [74]. The PICRUSt2 analysis in the present study showed the significant upregulation of pathways involved in the biosynthesis of the SCFA propionate, specifically pyruvate fermentation to propanoate I and L-glutamate degradation VIII to propanoate, in the healthy control group. Propionate, a three-carbon short-chain fatty acid (SCFA), is generated during microbial fermentation of dietary fibers by gut microbiota such as *Eubacterium hallii* [75,76], recently reclassified as *Anaerobutyricum hallii*, an anaerobic, Gram-positive bacterium within the *Lachnospiraceae* family [77]. *Eubacterium hallii* exhibits versatility in fermenting various substrates, producing key metabolites like butyrate and propionate, which are crucial for gut health, metabolic functions, and immune modulation [78,79]. Utilizing the acrylate and succinate-propionate pathways, this bacterium converts pyruvate and other substrates into propionate, essential for energy metabolism, glucose, and lipid regulation, and maintaining gut microbial balance [80]. The ability of propionate to cross the blood-brain barrier highlights its role in gut-brain axis communication and neurological function [69,81]. 

Reduced plasma SCFA levels, including propionate, in individuals with ADHD underscore the importance of *Eubacterium hallii* in SCFA production and interactions within the gut-brain axis [82,83]. In addition, the increased abundance of the *Eubacterium hallii* group in healthy controls may be related to gut-brain axis interactions via butyrate production. Butyrate, recognized for its anti-inflammatory and barrier-enhancing properties, functions as a histone deacetylase inhibitor [84]. In peripheral blood mononuclear cell (PBMC) models, butyrate effectively reduces pro-inflammatory cytokine production while promoting the release of anti-inflammatory cytokines [85,86]. Butyrate also influences T-cell differentiation through GPR109A signaling, fostering the development of regulatory T-cells and Interleukin-10 (IL-10)-producing T-cells [87]. Moreover, butyrate can enhance brain-derived neurotrophic factor (BDNF) production, encouraging neural synapse formation and differentiation, potentially improving brain health, and mitigating neurodegenerative conditions associated with ADHD [69,88]. Future research should explore the link between ADHD and these SCFAs, particularly propionate and butyrate, to assess their potential in modulating ADHD and preventing neurodevelopmental diseases.

To our knowledge, this is the first study on gut microbiome profiles in Thai ADHD children compared with healthy counterparts. The present study has several strengths. Firstly, we designed the investigation with an age- and sex-matched control condition to ameliorate age and gender heterogeneities. Secondly, we excluded factors interfering with gut microbiome profiles, such as obesity, allergic diseases, and atypical mental disorders. In addition, our participants had not used any medications or food supplements that might affect microbial diversity for at least two months before fecal collection. We actively gave consideration to limiting the magnitude of differences between groups from the onset of our analysis. However, we recognize that this study has certain limitations. It is a cross-sectional study conducted at a single time point in Thai children, which limits the generalizability of the findings to other ethnic populations and does not capture changes in the gut microbiome profile over time. Due to the global COVID-19 pandemic during sample collection, the small sample size is another limitation. Another one is that the widespread availability of the QIAamp^®^ DNA Stool Mini Kit in Thailand and the need for standardized procedures necessitated its use despite its known limitations. Notably, bead-beating intensity significantly influences the observed microbial community structure, giving results that correlate strongly with bacterial cell wall strength and improving DNA detection from Gram-positive bacteria. It is important to note that some gastrointestinal bacteria, particularly those in the Firmicutes phylum, have robust peptidoglycan layers, making them difficult to lyse without mechanical disruption [89]. Thus, the QIAamp^®^ Fast DNA Stool Mini Kit’s reliance on enzymatic digestion may lead to an underestimation of these bacteria. Future investigations will use a kit incorporating mechanical lysis, such as the QIAamp^®^ PowerFecal Pro DNA Kit from QIAGEN.

Furthermore, the 16S metagenomic profiling method itself has limitations as it does not adequately reflect the function or activity of the microbiota. To elucidate the functional roles of the intestinal microbiota and establish causal relationships between ADHD and the gut microbiome, metagenomic analysis combined with metabolomic profiling of individual samples is required. Additionally, the LEfSe data analysis method can be influenced by null inflation, where many zero values in relative abundance data may impact the outcomes. Alternative approaches, such as ANCOM-BC derived from ANCOM (Analysis of Composition of Microbiomes), have addressed this issue by integrating bias correction techniques, thereby reducing potential biases like false positives and null inflation. 

Regarding dietary analysis limitations, we employed semi-quantitative food frequency questionnaires (semi-FFQs) and 3-day food records without measuring plasma micronutrient levels. Additionally, there is a potential for underestimating dietary intake among study participants compared to reference values due to limitations in the dietary database. This method may not accurately assess the impact of nutritional metabolites on the gut microbiome, as FFQs might be less effective than plasma micronutrient measurements for evaluating dietary influences. We recommend that future research explore the association between plasma nutrients and the gut microbiome, highlighting the need for rigorously designed intervention trials to establish causality. Further studies should consider larger sample sizes, genetic variations, and biochemical pathways to support the current findings and elucidate the role of gut microbiota in ADHD manifestation and treatment.

## 5. Conclusions

The results of the present study suggest a potential link between the gut microbiome profiles and the clinical symptoms observed in treatment-naïve Thai children diagnosed with ADHD. There was a positive correlation between *CAG-352* and ADHD as well as a negative correlation between *the Eubacterium hallii* group and ADHD. Moreover, the intake of beta-carotene and vitamin B2 was associated with the abundance of particular gut microbiota in ADHD children. Subsequent investigations are warranted to validate these findings and to explore the influence of nutrients, therefore providing further insights into the mechanistic role of gut microbiota in ADHD.

## Figures and Tables

**Table 1:** Demographic data for 48 participants in this study.

Characteristics	ADHD (*n* = 24)	Healthy Children (*n* = 24)	*p*-Value ^†^
Gender, No. (%)			
Male	21 (87.5)	21 (87.5)	1.000
Female	3 (12.5)	3 (12.5)	
Age (year)	7 (7, 8)	7 (7,8)	0.887
Height (cm)	125.5 (120.5, 134.0)	125.0 (120.0, 132.5)	0.804
Body weight (kg)	24.6 (20.5, 28.0)	24.0 (20.5, 30.0)	0.702
BMI (kg/m^2^)	15.04 (14.59, 16.48)	15.72 (14.42, 19.10)	0.658
Gestational age (wk)	37 (37, 39)	39 (37.5, 39.5)	0.140
Birth weight (gm)	3156 (2735, 3465)	3155 (2945, 3415)	0.509
Birth length (cm)	50.5 (48.0, 55.0)	51.0 (50.1, 53.5)	0.860
Head circumference (cm)	33.0 (31.5, 34.0)	33.5 (33.0, 34.0)	0.373
6-month exclusive breastfeeding, No. (%)	7 (29.2)	5 (20.8)	0.706
Constipation, No. (%)	7 (29.2)	3 (12.5)	0.155
The SNAP-IV scale			
Inattention scores			
Parent	17.0 (15.0, 19.5)	8.0 (4.5, 9.0)	<0.001 *
Teacher	18.0 (16.0, 20.5)	6.0 (4.0, 9.0)	<0.001 *
Hyperactivity/impulsivity scores			
Parent	16.0 (11.5, 19.0)	4.0 (2.5, 8.0)	<0.001 *
Teacher	15.5 (12.0, 19.5)	4.0 (4.0, 5.0)	<0.001 *

Data are reported in number (%) and median (IQR, interquartile range). ^†^ The differences between groups were analyzed using the Chi-square test for categorical data or the Mann-Whitney U test for continuous data. * *p*-value < 0.05; *n*, number; ADHD, attention-deficit hyperactivity disorder; BMI, body mass index; SNAP-IV, Swanson, Nolan, and Pelham Rating Scale-IV.

**Table 2:** The association between the average scores on the inattention and hyperactivity/impulsivity subsets and the relative abundance of each genus in participants.

Genera	Inattention Subset Scoring				Hyperactivity/Impulsivity Subset Scoring			
	Parent		Teachers		Parent		Teachers	
	r_s_ ^†^	*p*-Value	r_s_ ^†^	*p*-Value	r_s_ ^†^	*p*-Value	r_s_ ^†^	*p*-Value
*Megamonas*	−0.14	0.004 *	−0.38	0.008 *	−0.40	0.005 *	−0.41	0.004 *
*Eubacterium hallii group*	−0.40	0.005 *	−0.44	0.002 *	−0.34	0.018 *	−0.41	0.004 *
*Enterobacter*	−0.19	0.193	−0.24	0.108	−0.09	0.531	−0.16	0.268
*Negativibacillus*	−0.31	0.031 *	−0.31	0.033 *	−0.17	0.248	−0.25	0.086
*Butyricimonas*	−0.27	0.065	−0.24	0.103	−0.32	0.025 *	−0.30	0.037 *
*Acidaminococcus*	−0.25	0.088	−0.24	0.107	−0.23	0.114	−0.24	0.102
*Succinivibrio*	0.19	0.193	0.31	0.030 *	0.25	0.09	0.29	0.043 *
*CAG-352*	0.34	0.020 *	0.40	0.005 *	0.37	0.009 *	0.36	0.012 *
*Alloprevotella*	0.39	0.006 *	0.32	0.026 *	0.20	0.185	0.15	0.305

^†^ The association was described as Spearman’s correlation coefficient (r_s_). * p-value < 0.05.

**Table 3:** Daily dietary intake analysis between ADHD patients and healthy children in this study.

Dietary Intake	DRIs (EAR/AI) in Children Aged 6–12 y	ADHD (*n* = 24)	Healthy Children (*n* = 24)	*p*-Value *
Energy (kcal)	1320–1800	1073 (859, 1353)	1150 (926, 1394)	0.578
Carbohydrate (CHO) (g)	ND	139 (108, 161)	142 (109, 168)	0.592
Protein (g)	24–40	50 (37, 62)	52 (39, 63)	0.635
Fat (g)	ND	39 (31, 54)	38 (35, 59)	0.578
CHO: Protein: Fat (%TEI)	ND	49: 19: 32	49: 19: 32	0.903
Dietary fiber (g)	ND	2.8 (1.8, 4.3)	3.6 (2.7, 5.0)	0.161
Sodium (mg)	325–1175	1254 (1005, 1667)	1455 (1239, 1908)	0.095
Adequate/Inadequate (n)		NA	NA	NA
Potassium (mg)	1625–3325	802 (649, 977)	963 (609, 1182)	0.386
Adequate/Inadequate (n)		NA	NA	NA
Calcium (mg)	800–1000	322 (176, 503)	414 (291, 631)	0.187
Adequate/Inadequate (n)		0/24	2/22	0.149
Phosphorus (mg)	500–1000	508 (353, 573)	523 (419, 730)	0.433
Adequate/Inadequate (n)		17/7	19/5	0.505
Iron (mg)	6.6–15.6	4.4 (3.5, 5.9)	4.8 (3.9, 6.6)	0.343
Adequate/Inadequate (n)		10/14	13/11	0.386
Copper (mg)	1.0–1.3	0.34 (0.25, 0.37)	0.39 (0.32, 0.48)	0.023 *
Adequate/Inadequate (n)		0/24	1/23	0.312
Magnesium (mg)	120–170	24 (14, 38)	18 (13, 40)	0.578
Adequate/Inadequate (n)		0/24	0/24	1.000
Selenium (mcg)	30–40	37 (22, 53)	34 (27, 45)	0.984
Adequate/Inadequate (n)		17/7	21/3	0.155
Zinc (mg)	6.3–9.5	3.2 (2.7, 4.2)	3.8 (2.8, 4.5)	0.174
Adequate/Inadequate (n)		7/17	9/15	0.540
Vitamin A (mcg)	350–550	154 (100, 284)	202 (176, 286)	0.257
Adequate/Inadequate (n)		6/18	6/18	1.000
Beta-carotene (mcg)	ND	68 (20, 307)	238 (89, 418)	0.039 *
Adequate/Inadequate (n)		NA	NA	NA
Vitamin B1 (mg)	0.6–0.9	0.8 (0.6, 1.4)	0.8 (0.6, 2.2)	0.386
Adequate/Inadequate (n)		19/5	21/3	0.439
Vitamin B2 (mg)	0.6–0.9	0.8 (0.6, 1.2)	1.1 (0.9, 1.5)	0.041 *
Adequate/Inadequate (n)		19/5	22/2	0.220
Vitamin B3 (mg)	8–12	9 (6, 15)	15 (10, 19)	0.022 *
Adequate/Inadequate (n)		19/5	23/1	0.081
Vitamin B6 (mg)	0.6–1	0.4 (0.3, 0.5)	0.4 (0.3, 0.4)	0.312
Adequate/Inadequate (n)		7/17	4/20	0.303
Vitamin B12 (mcg)	1.2–1.8	0.8 (0.3, 1.4)	0.6 (0.4, 1.1)	0.509
Adequate/Inadequate (n)		10/14	6/18	0.221
Vitamin C (mg)	40–60	7 (1, 21)	10 (6, 17)	0.293
Adequate/Inadequate (n)		5/19	5/19	1.000
Vitamin E (mg)	9–13	1.3 (0.8, 2.1)	1.1 (0.6, 1.9)	0.918
Adequate/Inadequate (n)		0/24	0/24	1.000

Data are reported in number (*n*) and median (IQR, interquartile range). * The differences between groups were analyzed using the Mann-Whitney U test for daily consumption and the Chi-square test for adequacy proportion, *p*-value < 0.05; NA, non-applicable; ND, not determined; ADHD, attention-deficit hyperactivity disorder; AI, adequate intake; DRIs, dietary reference intakes; EAR, estimated average requirement; TEI, total energy intake.

**Table 4:** The association between the dietary intake and the relative abundance of each genera.

Genera	Copper		Beta-Carotene		Vitamin B2		Vitamin B3	
	r_s_ ^†^	*p*-Value	r_s_ ^†^	*p*-Value	r_s_ ^†^	*p*-Value	r_s_ ^†^	*p*-Value
*Megamonas*	0.07	0.622	−0.03	0.843	−0.06	0.674	0.12	0.434
*Eubacterium hallii* group	0.20	0.180	0.36	0.013 *	0.12	0.405	0.21	0.146
*Enterobacter*	0.15	0.306	0.01	0.967	0.16	0.279	0.11	0.470
*Negativibacillus*	0.10	0.484	0.23	0.117	0.10	0.506	0.22	0.140
*Butyricimonas*	0.19	0.190	0.11	0.441	0.17	0.240	0.13	0.388
*Acidaminococcus*	−0.01	0.959	0.10	0.496	0.14	0.349	−0.03	0.816
*Succinivibrio*	−0.22	0.137	−0.31	0.035 *	−0.21	0.155	−0.16	0.280
*CAG-352*	−0.02	0.902	−0.20	0.170	−0.09	0.551	−0.13	0.386
*Alloprevotella*	−0.24	0.104	−0.21	0.159	−0.32	0.026 *	−0.22	0.137

^†^ The association was described as Spearman’s correlation coefficient (r_s_). * p-value < 0.05.

## Data Availability

The datasets presented in this study were uploaded to the Sequence Read Archive of the National Center for Biotechnology Information (NCBI) with the Bioproject accession number PRJNA988940 and are available on request from the corresponding author.
